# Single-Walled Carbon Nanotubes with Red Phosphorus in Lithium-Ion Batteries: Effect of Surface and Encapsulated Phosphorus

**DOI:** 10.3390/nano13010153

**Published:** 2022-12-29

**Authors:** Anna A. Vorfolomeeva, Svetlana G. Stolyarova, Igor P. Asanov, Elena V. Shlyakhova, Pavel E. Plyusnin, Evgeny A. Maksimovskiy, Evgeny Yu. Gerasimov, Andrey L. Chuvilin, Alexander V. Okotrub, Lyubov G. Bulusheva

**Affiliations:** 1 Nikolaev Institute of Inorganic Chemistry SB RAS, 3 Acad. Lavrentiev Ave., 630090 Novosibirsk, Russia; 2 Boreskov Institute of Catalysis, SB RAS, 5 Acad. Lavrentiv Ave., 630090 Novosibirsk, Russia; 3 CIC NanoGUNE BRTA, Tolosa Hiribidea 76, E-20018 Donostia-San Sebastián, Spain; 4 IKERBASQUE, Basque Foundation of Science, Maria Diaz de Haro 3, E-48013 Bilbao, Spain

**Keywords:** carbon nanotubes, phosphorus, encapsulation, lithium-ion batteries

## Abstract

Single-walled carbon nanotubes (SWCNTs) with their high surface area, electrical conductivity, mechanical strength and elasticity are an ideal component for the development of composite electrode materials for batteries. Red phosphorus has a very high theoretical capacity with respect to lithium, but has poor conductivity and expends considerably as a result of the reaction with lithium ions. In this work, we compare the electrochemical performance of commercial SWCNTs with red phosphorus deposited on the outer surface of nanotubes and/or encapsulated in internal channels of nanotubes in lithium-ion batteries. External phosphorus, condensed from vapors, is easily oxidized upon contact with the environment and only the un-oxidized phosphorus cores participate in electrochemical reactions. The support of the SWCNT network ensures a stable long-term cycling for these phosphorus particles. The tubular space inside the SWCNTs stimulate the formation of chain phosphorus structures. The chains reversibly interact with lithium ions and provide a specific capacity of 1545 mAh·g^−1^ (calculated on the mass of phosphorus in the sample) at a current density of 0.1 A·g^−1^. As compared to the sample containing external phosphorus, SWCNTs with encapsulated phosphorus demonstrate higher reaction rates and a slight loss of initial capacity (~7%) on the 1000th cycle at 5 A·g^−1^.

## 1. Introduction

Elementary phosphorus (P) has attracted considerable attention as a promising anode material for lithium-ion batteries (LIBs) due to the strong affinity between P and the metal ion [1]. Phosphorus in the course of electrochemical reactions is able to accept three lithium ions (Li_3_P) [2], which provide a theoretical capacity of 2596 mAh·g^−1^ [3]. Among all allotropes, red phosphorus is commercially available, chemically stabile and environmentally friendly [4].

Limitations in the use of red phosphorus in LIBs are associated with its low electrical conductivity (~10^−14^ S·cm^−1^) [5,6], which leads to slow electrochemical redox reactions and poor rate performance. In addition, the high initial capacity of phosphorus drops rapidly during charge-discharge due to volume expansion (>300%) with the attachment of lithium [7]. As a result, red phosphorus anodes exhibit a rapid capacity loss, low Coulombic efficiency and electrode deterioration during cycling [8,9,10].

To alleviate these problems, a carbon matrix is used [1]. Allotropes of carbon are generally hydrophobic and resistant to chemicals under normal conditions. The combination of phosphorus and carbon is an opportunity to obtain a conductive material in which the carbon protects and/or stabilizes phosphorus during operation of the device [11]. Most of these combinations are obtained either by deposition of phosphorus on carbon using the evaporation-condensation technique [4,11,12,13,14,15,16], or by simple mixing of the components in a ball mill [2,17,18,19,20,21,22,23]. However, in this case, the carbon matrix is able to prevent material degradation only for a short time of LIB operation (less than 100 cycles) [24,25].

At present, it is generally accepted that phosphorus-based anodes should be made in the form of nanocomposites, in which the phosphorus component is finely dispersed in carbon [21]. Such materials are porous (micro- or mesoporous) carbon [3,4,13], modified graphene [6,26,27], carbon nanofibers [28] and carbon nanotubes (CNTs) [2,20,29]. Besides, it is very important to maintain a good electrical connection between the phosphorus and the conductive matrix during the lithiation processes.

The combination of phosphorus with porous carbon has been shown to provide good cycling stability of the composite electrode due to pores that prevent phosphorus sputtering [30]. Channels between CNTs and inside them can also perform this phosphorus-stabilizing role [31], preventing the contact of phosphorus with environmental molecules. CNTs are characterized by high conductivity (>10^2^ S·cm^−1^) [32], mechanical strength (Young’s modulus < 1 TPa) [33,34,35], tensile strength < 70 GPa [35,36]) and flexibility [37,38]. Currently, several studies are reported using red phosphorus/CNT composites to improve electrochemical performance in lithiation/delithiation processes [17,39,40]. A high Coulombic efficiency in the fiftieth cycle was achieved for phosphorus-encapsulated CNTs; however, the specific capacity during cycling significantly decreased [41].

It can be assumed that the structure of phosphorus affects its interaction with lithium ions, although there are no clear studies in this area. The arrangement of phosphorus atoms inside the nanotube depends on the size of the internal space [42,43,44,45] and the synthesis conditions. The vaporization-condensation process is commonly used to fill CNTs with phosphorus [29,41,44]. This process also leads to the deposition of phosphorus on the outer surface of CNTs [46,47,48]. Phosphorus oxides, formed when the deposit comes into contact with laboratory air, are easily washed off with ethanol [43] or CS_2_ [5,48], but the core of red phosphorus nanoparticles is quite stable. During LIB operation, this external phosphorus can detach from the conductive carbon surface and expand uncontrollably [44]. How to mitigate or suppress these negative effects is the key to improving battery performance [39].

Here, we study the effect of phosphorus located on the surface of SWCNTs and/or inside the channels of SWCNTs on the performance of the composite electrode in LIBs. Commercial SWCNTs (trademark Tuball) 1.6–2.9 nm in diameter with closed or open ends were used as a substrate for red phosphorus condensation at 800 °C. The structure and composition of the synthesized nanomaterials were determined by scanning electron microscopy (SEM) combined with elemental mapping by energy-dispersive X-ray spectroscopy (EDS), high-angle annular dark-field scanning transmission electron microscopy (HAADF-STEM), powder X-ray diffraction (XRD), Raman spectroscopy, thermogravimetric analysis (TGA) and X-ray photoelectron spectroscopy (XPS). The nanomaterials were comparatively studied in LIBs using galvanostatic discharge-charge (GDC) and cyclic voltammetry (CV) measurements. SWCNTs with the encapsulated phosphorus showed the best rate and cycling performance as well as faster kinetics of redox reactions as compared to SWCNTs with external phosphorus deposits.

## 2. Materials and Methods

### 2.1. Synthesis

SWCNTs were purchased from OCSiAl (Novosibirsk, Russia). Treatment with concentrated HCl was used to remove the accessible iron catalyst from the sample and the resulting SWCNTs served as a substrate to form the external phosphorus deposits (sample SWCNTs/P). To open the caps of the nanotubes, the original sample was heated in air at 500 °C for 1 h and then washed with concentrated HCl and dried in air. The obtained sample is labeled as o-SWCNTs and used as a reference.

Synthesis of composite nanomaterials was carried out in an H-shaped quartz ampoule by the vaporization-condensation method using amorphous red phosphorus (Prime Chemicals Group, >98%). A phosphorus sample (60 mg) and a nanotube sample (30 mg) were placed in different parts of an H-shaped quartz ampoule, the ampoule was pumped out, sealed, heated in a muffle furnace to 800 °C with a rate of ~7 °C·min^−1^ and kept at the final temperature for 48 h. After that, the ampoule was naturally cooled to room temperature and carefully opened in air. The product obtained with o-SWCNTs is designated as P@SWCNTs/P. Treatment with an aqueous NaOH solution (2.5 M) was used to remove external phosphorus deposits [46]. Briefly, a P@SWCNTs/P sample was vigorously stirred at 60 °C for 5 h, filtered, washed to neutral pH and dried in an oven. This sample is designated P@SWCNTs. A reference sample of red phosphorus P_re_ was synthesized by recrystallization of a commercial amorphous phosphorus sample under the conditions used for the synthesis of composite nanomaterials.

### 2.2. Instrumental Methods

The sample morphology was characterized by SEM on a JEOL JSM 6700 F (JEOL LTD., Tokyo, Japan) microscope. Elemental analysis was carried out by EDS on a Bruker XFlash 6 spectrometer. HAADF-STEM and EDS mapping were carried out at 80 kV on a Themis Z microscope (Thermo Fisher Scientific Inc., Eindhoven, The Netherlands) equipped with x-FEG monochromator, dual side C_s_ spherical aberration correction and Super-X EDS detector. High-resolution TEM (HRTEM) images were obtained using a Titan 60–300 microscope (FEI, Eindhoven, The Netherlands) at an accelerating voltage of 80 kV.

Raman spectra were collected on a LabRAM HR Evolution Horiba spectrometer (Horiba, Kyoto, Japan) using excitation from an Ar^+^ laser at 514 nm at a power of 1 mW. The spectra at room temperature were obtained in the backscattering geometry. The laser beam was focused to a diameter of 2 μm using an LMPlan FL 50×/0.50 Olympus objective. The spectral resolution was 3.0 cm^−1^. The size of the laser spot was 1 μm. The XRD patterns of the samples were obtained on a Shimadzu XRD-7000 diffractometer (Shimadzu Europa GmbH, Duisburg, Germany) using Cu Kα radiation and Ni filter on the reflected beam.

Simultaneous thermal analysis (STA) includes thermogravimetric (TG) and evolved gas analysis using mass spectrometry. Measurements were performed on a NETZSCH STA 449F1 Jupiter instrument (Selb/Bayern, Germany). The sample was placed in an open Al_2_O_3_ crucible and heated in a helium flow (20 mL·min^−1^) from room temperature to 1000 °C at a rate of 10 °C·min^−1^. An electron impact ionizer operated at an energy of 70 eV. The ion currents of the selected mass/charge (*m*/*z*) numbers were monitored in multiple-ion detection mode with a collection time of 0.1 s for each channel.

XPS measurements were carried out on an X-ray photoelectron spectrometer FleXPS (Specs GmbH, Berlin, Germany) via an electron energy analyzer Phoibos 150 with a delay line detector (DLD) electron detector. The spectra were excited by Al Kα radiation (1486.7 eV). The transmission energy of the analyzer was 20 eV. The vacuum in an analytical chamber was ~10^−9^ mbar. Elemental composition was evaluated from the ratio of the areas under corresponding core-level peaks of the survey spectra, taking into account the photoionization cross sections at a given photon energy. Data processing was carried out using the CasaXPS package (Casa Software Ltd., Teignmouth, UK). The fitting of the spectra was performed using symmetric lines as a product of the Gaussian and Lorentzian components. The XPS P 2p spectrum was fitted by two spin–orbit P 2p_3/2_–P 2p_1/2_ doublets with a ratio of the components of 2:1 and separation of 0.84 eV after subtraction of the background signal by Shirley’s method.

### 2.3. Electrochemical Measurements

To prepare the electrode material, a portion of the testing sample (~30 mg) was mixed with polyvinylidene fluoride (PVDF-2, 10 wt%), necessary for better adhesion to the copper foil surface, conductive carbon additive super P (10 wt%) and 2–3 mL of N-methyl-pyrrolidone solvent. The mixture was stirred with steel balls on a vibrating mixer for 1 h. The resulting suspension was distributed over a copper foil and dried at 80 °C for 12 h under vacuum. Electrodes with a diameter of 10 mm were obtained using a cylindrical cutter. The mass of the active electrode material was determined from the mass difference between the initial copper substrate and the substrate with the deposited material. The weight of each electrode material was ca. 0.4–0.5 mg. The electrochemical cells were assembled in a glove box filled with argon with the water and oxygen content less than 0.1 ppm. The CR2032 coin cells were assembled with lithium metal as the counter electrode. The electrolyte was a 1 M solution of LiPF_6_ in a mixture of ethylene carbonate and dimethyl carbonate (1:1 by volume) from Merck Co. GDC tests were performed on a NEWARE CT-3008 station (Neware Technology Ltd., Shenzhen, China) in the voltage range from 0.01 to 2.50 V vs. Li/Li^+^ at current densities from 0.1 A·g^−1^ to 5·A g^−1^. CV measurements were carried out using a Bio-Logic SP-300 station (Bio-Logic Science Instrument, Seyssinet-Parist, France) from 0.01 to 2.5 V at scan rates varied from 0.1 to 1 mV·s^−1^.

## 3. Results

### 3.1. Characterization of Nanomaterials

**Figure 1:**
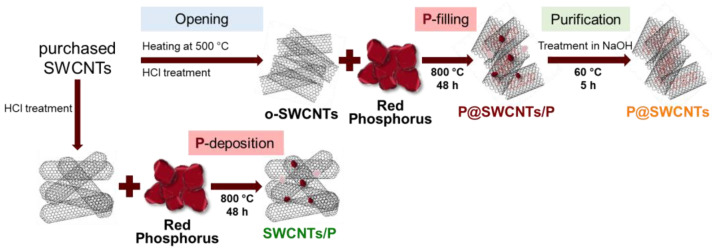
Schematic illustration of the synthesis of composite nanomaterials from SWCNTs and red phosphorus.

The synthesis of composite nanomaterials is illustrated in Figure 1. At high temperatures, phosphorus evaporates and moves into the part containing carbon nanotubes. Vapors penetrate into the cavities of nanotubes when their caps are opened (o-SWCNTs sample). Upon cooling, phosphorus species condense on accessible substrates, in particularly, on the inner and outer surfaces of nanotubes and the ampoule walls. Therefore, the P@SWCNTs/P sample contains encapsulated phosphorus and phosphorus covering the nanotubes. Treatment of this sample with NaOH removes external phosphorus [46] and the resulting P@SWCNTs are nanotubes filled with phosphorus. In the SWCNTs/P sample, phosphorus should be present only on the outer surface of the nanotubes, because their ends are closed.

**Figure 2:**
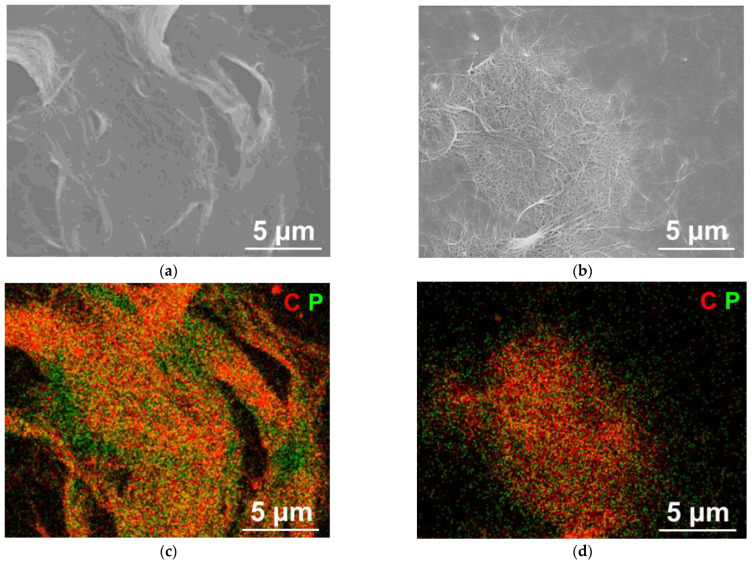
(**a**,**b**) SEM images and (**c**,**d**) corresponding EDS combined maps of carbon and phosphorus elements for (**a**,**c**) P@SWCNTs/P and (**b**,**d**) P@SWCNTs.

The SEM study shows that the P@SWCNTs/P sample has a fibrous structure; however, individual bundles of nanotubes can be distinguished only at breaks (Figure 2a). This is due to the coating of nanotubes with phosphorus. The bundles of nanotubes are clearly visible in the P@SWCNTs image (Figure 2b), which confirms effectiveness of the purification procedure used. EDS mapping shows differences between P@SWCNTs/P (Figure 2c) and P@SWCNTs (Figure 2d). In the former image, there are areas of phosphorus accumulation, which may correspond to surface phosphorus nanoparticles. In the latter image, phosphorus is evenly distributed throughout the sample.

The change in the structure of the initial o-SWCNTs sample after phosphorus condensation on the outer and inner surfaces of the nanotube is supported by XRD analysis (Figure S1). The o-SWCNTs pattern contains reflections from bundled nanotubes [49]. These reflections are present in the P@SWCNTs/P and P@SWCNTs patterns and overlap with broad reflections at approximately 2θ of 18°, 24° and 32°, corresponding to fibrous red phosphorus [50].

**Figure 3:**
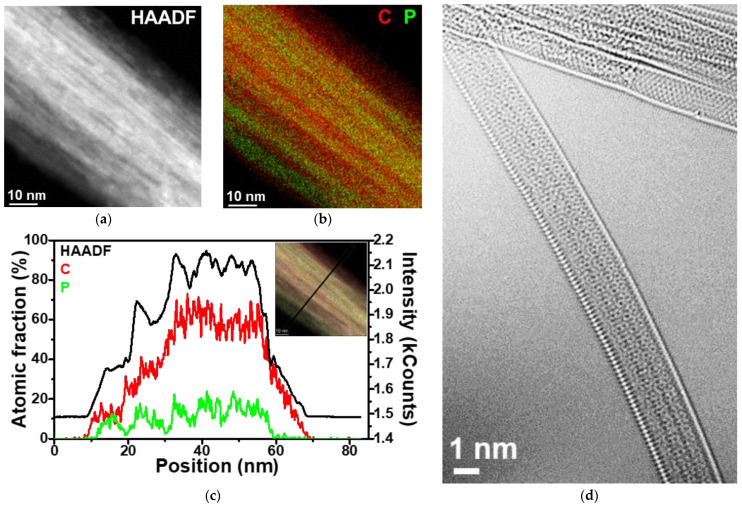
(**a**) HAADF image of P@SWCNTs; (**b**) corresponding EDS elemental map of carbon and phosphorus and (**c**) distribution of elements perpendicularly to the nanotube bundle (along the line shown in the inset); (**d**) HRTEM image of phosphorus-filled SWCNTs.

To confirm the filling of nanotubes, we performed a study of P@SWCNTs using HAADF/STEM and EDS analysis. Figure 3a shows a HAADF image of a nanotube bundle. Bright strips along the axis of the bundle correspond to phosphorus since this element is heavier than carbon. The combined elemental map of phosphorus and carbon is depicted in Figure 3b. The phosphorus signal (green) appears evenly along the length of the bundle, while in the transverse direction it alternates with the carbon signal (red). This distribution of phosphorus indicates its location in the internal cavities of nanotubes or between neighboring nanotubes.

EDS analysis perpendicular to the bundle axis (inset in Figure 3c) found a low fraction of phosphorus (Figure 3c). The phosphorus content determined from the EDS data (Figure S2) is ~6 at% (15 wt%). The fact that the carbon profile is wider (Figure 3c) confirms the absence of phosphorus on the surface of the SWCNT bundle. The phosphorus profile is periodic due to the restrictive effect of the carbon walls. The lower intensity in the phosphorus signal is accompanied by a peak in the carbon signal. Therefore, phosphorus has a preferable location along the nanotubes. The HRTEM examination revealed the encapsulation of phosphorus in SWCNTs (Figure 3d). Phosphorus forms long ordered chains similar to the fibrous structure of red phosphorus [42].

The phosphorus content determined from the analysis of the XPS survey spectra is ca. 16 at% (32 wt%) in P@SWCNTs/P, ca. 11 at% (23 wt%) in SWCNTs/P and ca. 9 at% (18 wt%) in P@SWCNTs (Figure S3a). The latter value agrees well with the phosphorus content obtained using EDS (Figure S2). The same ratio of reagents was used in all synthesis and the lower content of phosphorus in SWCNTs/P, where the nanotube caps are closed, as compared to P@SWCNTs/P, where the nanotubes are open, indicates that a surface is required for phosphorus condensation. During the synthesis of SWCNTs/P, only the outer surface of the nanotubes is accessible and the residual phosphorus is deposited on the walls of the ampoule.

**Figure 4:**
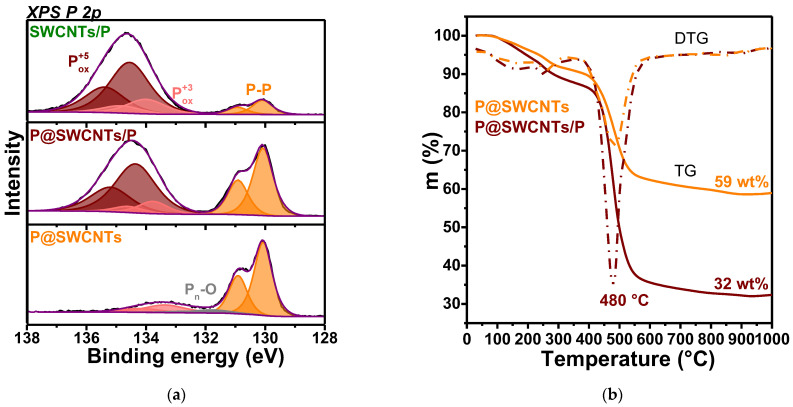
(**a**) XPS P 2p spectra of P@SWCNTs (encapsulated phosphorus), P@SWCNTs/P (encapsulated and external phosphorus) and SWCNTs/P (external phosphorus); (**b**) TG and DTG curves measured for P@SWCNTs/P and P@SWCNTs in helium.

The XPS P 2p spectra of the composite samples were fitted by three spin-orbit doublets (Figure 4a). The low-energy doublet with P 2p_3/2_ binding energy of 130.1 eV is assigned to elementary phosphorus, i.e., P-P species. This component dominates in the P@SWCNTs spectrum and has the lowest relative intensity in the SWCNTs/P spectrum. The P 2p_3/2_ line at 133.8 eV is associated with the P^+3^ state in oxidized species [46,51]. For a good fitting of the P@SWCNTs spectrum, an additional weak doublet was added between the P-P and P^+3^ components. The P 2p_3/2_ binding energy of 132.0 eV corresponds to the oxygen-terminated ends of phosphorus polymers [52]. We attribute this state to the ends of the encapsulated phosphorus chains, which attached to oxygen when the sample came into contact with laboratory air.

The P 2p spectra of P@SWCNTs/P and SWCNTs/P samples contain an intense doublet with the P 2p_3/2_ binding energy of 134.4 eV from the P^+5^ state in oxidized forms [51,53]. Doubles of the oxidized P^+3^ and P^+5^ states form a peak with a maximum at 134.5 eV and this peak is considerably higher in the SWCNTs/P spectrum (Figure S3b). The ratio of the area of this peak to the area under the P-P doublet is 8.8 for SWCNTs/P and 3.5 for P@SWCNTs/P. The outer surface of the SWCNTs can be covered by white phosphorus (this form is clearly visible in the ampoule after synthesis) and red phosphorus nanoparticles, observed by SEM/EDS analysis (Figure 2a,c). White phosphorus and the surface of phosphorus nanoparticles are oxidized when interacting with oxygen and water molecules from the air, while the nanotube walls protect the encapsulated phosphorus. The absence of high-energy doublet in the P 2p spectrum of P@SWCNTs (Figure 4a) confirms the cleaning of the outer surface of the nanotubes in this sample.

The decomposition of phosphorus-filled samples P@SWCNTs/P and P@SWCNTs was studied in helium by the STA method (Figure 4b). The broad peak observed in the differential TG (DTG) curves between 40–200 °C corresponds to removal of adsorbed water molecules. Next peak at ~250 °C is accompanied by the release of P, PH_2_ and PH_3_ species from the samples (Figure S4). This process causes a loss of ~6 wt% for P@SWCNTs/P and ~5 wt% for P@SWCNTs. The main weight loss occurs at ~480 °C due to the evaporation of P, PO and P_2_. As a result of this process P@SWCNTs/P and P@SWCNTs samples lose ~51 wt% and ~28 wt%, respectively. The SWCNTs/P sample shows a similar thermal behavior (Figure S5) and has a mass loss of ca. 27 wt% over the range from 380 to 600 °C. The loss of samples stops after ~850 °C and the mass of the residue corresponds to the weight of SWCNTs in the composite. The almost two times lower SWCNT mass in the P@SWCNTs/P sample as compares to the P@SWCNTs sample (Figure 4b) is due to the fact that the former sample contains external phosphorus.

**Figure 5:**
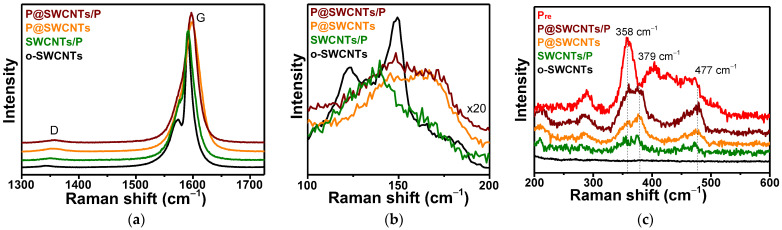
Raman spectra of o-SWCNTs sample and composite nanomaterials: (**a**) the first order region and (**b**) the RBM-mode region, presented with a zoom of 20; (**c**) Raman scattering from 200 to 600 cm^−1^ for reference o-SWCNTs and recrystallized phosphorus (P_re_) samples and composite nanomaterials.

Raman spectroscopy is used to compare the structure of SWCNTs and phosphorus in reference and composite samples. The first-order region of the o-SWCNTs spectrum exhibits a weak D-band at 1357 cm^−1^ (Figure 5a) due to the low density of defects in the nanotube walls. Since the intensity of this band increases insignificantly in the spectra of composite nanomaterials, we conclude that carbon atoms do not form covalent bonds with phosphorus. The G-band at 1591 cm^−1^ with a shoulder at 1574 cm^−1^ observed in the Raman spectrum of o-SWCNTs corresponds to longitudinal optical and transverse optical phonons in semiconducting nanotubes [54]. The shoulder becomes less pronounced in SWCNTs/P and overlaps with the main G-band peak in P@SWCNTs and P@SWCNTs/P. Thus, the nanotube surface is modified in composite nanomaterials as compared to the initial o-SWCNTs. Interestingly the G-band is upshifted by ~7 cm^−1^ in the spectra of P@SWCNTs and P@SWCNTs/P. This p-doping of SWCNTs is not observed for the SWCNTs/P sample, where phosphorus located on the outer surface of the nanotubes. Therefore, only the encapsulated phosphorus accepts electron density from SWCNTs. According to density functional calculations, this occurs when phosphorus has a chain-like structure [46].

The peaks that appeared in the Raman spectrum of o-SWCNTs at 123 and 148 cm^−1^ (Figure 5b) correspond to the radial breathing modes (RBM) [55]. This region changes strongly in the spectra of composite nanomaterials. The radial vibrations of the SWCNTs are limited by the phosphorus coating in the SWCNTs/P sample. In the case of P@SWCNTs, upshifts of the peaks can be associated with p-doping, which changes the conditions for resonant excitation of SWCNTs [56]. Both the above effects are valid for P@SWCNTs/P, whose spectrum is a superposition of the spectra of SWCNTs/P and P@SWCNTs.

Raman scattering in phosphorous structures gives peaks in the region of 200–600 cm^−1^ [57], where the spectrum of o-SWCNTs has no features (Figure 5c). The Raman spectrum of phosphorus condensed in an ampoule without SWCNTs (sample P_re_) exhibits a set of peaks characteristics of amorphous red phosphorus [58]. The SWCNT substrate significantly modifies the structure of phosphorus. The spectra of composite nanomaterials show high intensity at ~379 cm^−1^, where the P_re_ spectrum has a gap in intensity and suppressed scattering in the range of 390–446 cm^−1^. The Raman spectrum of P@SWCNTs is very close to the spectra of red phosphorus with the Hittorf’s structure [59] and fibrous structure [60]. Both of these phosphorus crystals consist of polymeric tubes with a pentagonal cross section of P_8_ and P_9_ cages connected by P_2_ bridges [61]. The P@SWCNTs/P spectrum looks like a combination of the P_re_ and P@SWCNTs spectra. Indeed, the sum of these spectra in a ratio of 0.3 to 1.1 gives a line that agrees well with the experimental spectrum of P@SWCNTs/P (Figure S6). The Raman spectrum of the SWCNTs/P sample has a low intensity of P-P vibrations due to the small size of elementary phosphorus cores as shown by XPS data (Figure 4a).

### 3.2. Electrochemical Properties

**Figure 6:**
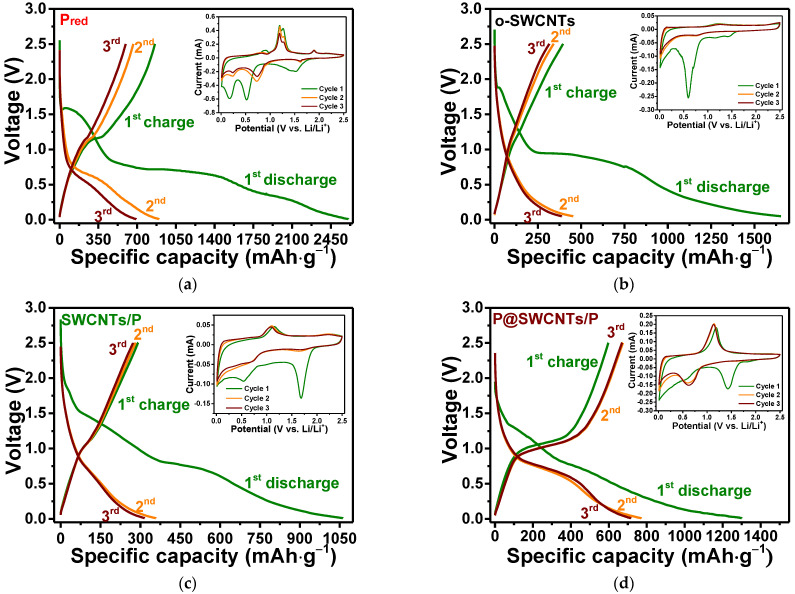

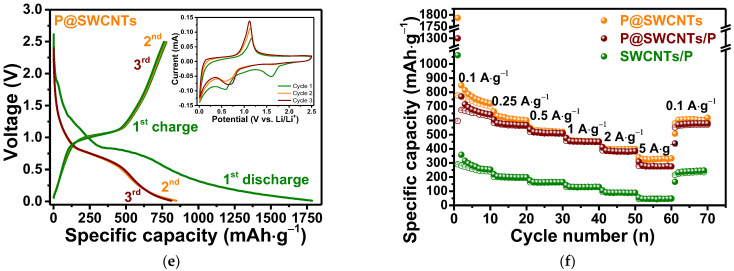
First three GDC curves with insets of the first three CV curves measured for (**a**) red phosphorus (P_red_), (**b**) o-SWCNTs, (**c**) SWCNTs/P, (**d**) P@SWCNTs/P and (**e**) P@SWCNTs; (**f**) Rate capability of composite nanomaterials.

Figure 6a–e compare the first three GDC curves measured for amorphous red phosphorus, open SWCNTs and three composite materials at a current density of 0.1 A·g^−1^. The first discharge and charge capacities of 2607 and 861 mAh·g^−1^ for red phosphorus (Figure 6a) give a very low initial Coulombic efficiency (ICE) of ~33%. A sufficient capacity drop is observed in the following second and third cycles. This is due to the low electrical conductivity of amorphous red phosphorus and the large change in its volume during lithiation. The o-SWCNTs reference sample also shows a low ICE of ~24% (Figure 6b). However, the second and third discharge-charge curves practically coincide. The large loss of capacity of o-SWCNTs in the first cycle is mainly attributed to the formation of a solid-electrolyte interphase (SEI) layer on the electrode surface, which is observed as a long plateau at ~0.9 eV during the first discharge and the irreversible incorporation of lithium into the carbon matrix [62,63].

The first discharge and charge capacities are 1062 and 291 mAh·g^−1^ for SWCNTs/P (Figure 6c), 1299 and 597 mAh·g^−1^ for P@SWCNTs/P (Figure 6d) and 1787and 779 mAh·g^−1^ for P@SWCNTs (Figure 6e). The highest value of the initial capacity of the P@SWCNTs sample can be related to two factors. First, the nanotubes in this composite are free from phosphorus coating and, therefore, lithium can accumulate in the space between them. Second, the external phosphorus in SWCNTs/P and P@SWCNTs/P is significantly oxidized as shown by the XPS P 2p spectra (Figure 4a). This oxidized phosphorus does not participate in electrochemical reactions and, in addition, can prevent the penetration of lithium ions to the elementary phosphorus core.

The ICE is 27% for SWCNTs/P, 46% for P@SWCNTs/P and 44% for P@SWCNTs. As follows from the GDC curves measured for reference samples (Figure 6a,b), both components of composite nanomaterials are responsible for the high irreversible capacity of the first cycle. It is very important to maintain good electrical contact between the phosphorus and the carbon matrix in order to maintain charge transfer [10]. Phosphorus deposition only on the outer surface of SWCNTs (SWCNTs/P sample) does not solve the problem of high irreversible capacity. However, the encapsulation of phosphorus into the internal channels of nanotubes increases the ICE value and leads to an insignificant change in the GDC curves in subsequent cycles.

CV measurements were used to determine the potentials of electrochemical processes occurring with electrode materials. The first cathodic curves for all electrodes show a set of peaks in the potential range of 0.17–1.7 V. A wide peak at ca. 1.5 V and two peaks between 0.5 and 0.17 V observed for red phosphorus (inset in Figure 6a) refer to the process of activation of the incorporation of lithium ions into phosphorus [2]. The first cathodic curve of o-SWCNTs (inset in Figure 6b) exhibits a peak at about 0.6 V due to the formation of the SEI layer [64]. This peak is also observed for all composite nanomaterials, but with a lower intensity, which indicates that the modification of the SWCNT surface as a result of the synthesis of composite nanomaterials. Irreversible and broadened cathodic peaks at 1.4–1.7 V (insets in Figure 6c–e) can be associated with both the activation of the phosphorus component and the irreversible loss of phosphorus in the SEI layer. Remarkably, the intensity of this peak decreases as the external oxidized phosphorus decreases from SWCNTs/P to P@SWCNTs/P and then to the P@SWCNTs sample.

The second and third CV curves of composite nanomaterials show two reversible redox peaks. The cathodic peak at ~0.62–0.69 V and the corresponding anodic peak at ~1.08–1.13 V (insets in Figure 6c–e) correspond to the reversible reaction P + xLi^+^ + xe^−^ ↔ Li_x_P [11] with stepwise formation of LiP, Li_2_P and Li_3_P [3,11]. On the second and third GDC curves, these processes form plateaus, which are more repetitive during P@SWCNTs cycling (Figure 6e). The redox pair at 0.01/0.15 V vs. Li/Li^+^ corresponds to the insertion/extraction of lithium ions into/from the SWCNT component [65].

Figure 6f compares the rate performance of composite nanomaterials for ten cycles at each applied current density. The specific capacity is calculated based on the weight of the electrode material. The SWCNTs/P sample showed the lowest values, in particular, 256, 200, 164, 130, 89 and 47 mAh·g^−1^ at current densities of 0.1, 0.25, 0.5, 1, 2 and 5 A·g^−1^, respectively. When the current density returned to 0.1 A·g^−1^, the SWCNTs/P electrode was still able to maintain a delithiation capacity of 245 mAh·g^−1^. Notably, these values exceed the capacity values of red phosphorus by 20–55% (Figure S7) at current densities above 0.5 A·g^−1^. At high cycling rates, the rate of charge transfer becomes important. We assume that in our case this effect is achieved due to the conducting property of SWCNTs. The P@SWCNTs sample showed the best performance with capacities of 734, 605, 520, 452, 398 and 328 mAh·g^−1^ at current densities of 0.1, 0.25, 0.5, 1, 2 and 5 A·g^−1^, respectively (Figure 6f). There was no difference between delithiation and lithiation values, indicating good electrode stability. After sixty test cycles, the electrode delivered 609 mAh·g^−1^ at 0.1 A·g^−1^. The P@SWCNTs/P electrode showed a lower capacity (by 40–90 mAh·g^−1^) than the P@SWCNTs electrode at 0.1, 0.25 A·g^−1^ and 5 A·g^−1^ and comparable capacity at a current density of 0.5 to 2 A·g^−1^. The capacity of this electrode was 579 mAh·g^−1^ at following cycles at 0.1 A·g^−1^. At all applied current densities, the specific capacity of P@SWCNTs and P@SWCNTs/P exceeds the sum of the capacities of the individual components (Figure S7).

**Figure 7:**
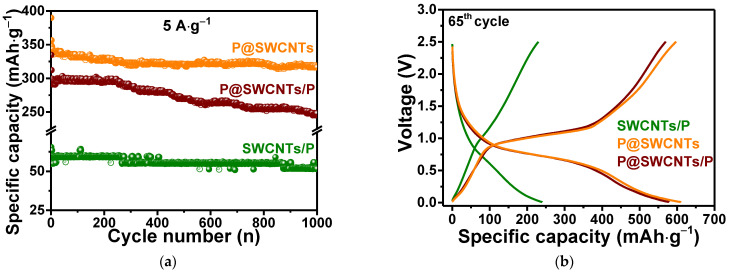
(**a**) Long-term cycling of composite nanomaterials at 5 A·g^−1^ and (**b**) GDC curves measured at 65th cycle at a current density of 0.1 A·g^−1^.

Long-term cycling tests of composite nanomaterials were carried out at a current density of 5 A·g^−1^. The specific capacity of SWCNTs/P continuously decreased (Figure 7a). However, after one thousand cycles, the electrode loses only ~12% of its initial capacity. The P@SWCNTs/P electrode maintained a capacity of ~296 mAh·g^−1^ for ~250 cycles, after which the capacity gradually decreased to 248 mAh·g^−1^ at 1000th cycle. The capacity loss for this electrode was ~16%. The P@SWCNTs electrode showed a slight decrease in capacity from 340 to 317 mAh·g^−1^ during cycling, which corresponds to a capacity loss of ~7%.

The processes affecting the capacity of composite nanomaterials can be determined from the GDC curves measured after long-term cycling (Figure 7b). The P@SWCNTs/P and P@SWCNTs curves exhibit extended plateaus corresponding to reversible reactions between phosphorus and lithium ions. The contribution of the SWCNT component to the capacity is greater for P@SWCNTs. In this case, the outer surface of the SWCNTs is not contaminated with phosphorus and lithium ions can intercalate between the nanotubes. Sufficiently stable operation of all composite nanomaterials in the electrochemical cells is most likely associated with the formation of a conducting network by intertwined nanotubes.

**Figure 8:**
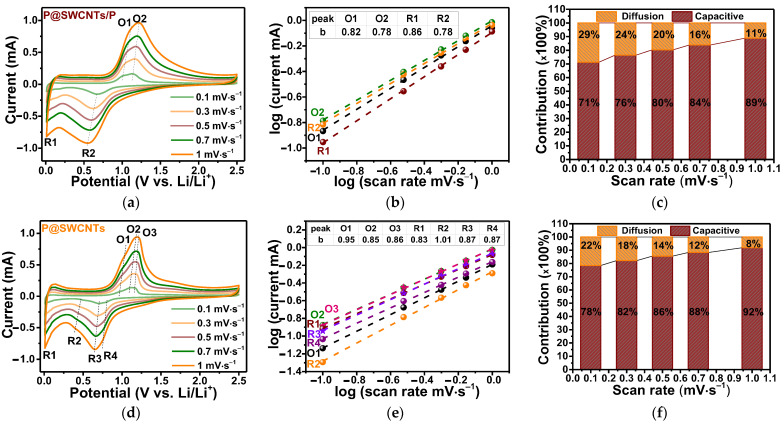
CV curves of (**a**) P@SWCNTs/P and (**d**) P@SWCNTs nanomaterials at different scan rates; log(*i*) vs. log(*v*) plots for selected peaks of (**b**) P@SWCNTs/P and (**e**) P@SWCNTs; the normalized ratio of capacitive and diffusion-controlled contributions at different scan rates of (**c**) P@SWCNTs/P and (**f**) P@SWCNTs.

To understand the higher rate performance of P@SWCNTs as compared to P@SWCNTs/P (Figure 7a), a kinetic analyses of the electrochemical processes was carried out. Figure 8 compares data obtained at scan rates of 0.1, 0.3, 0.5, 0.7 and 1.0 mV·s^−1^. The CV curves show two reduction peaks and two oxidation peaks for the P@SWCNTs/P electrode (Figure 8a), while four reduction peaks and three oxidation peaks are detected for the P@SWCNTs electrode (Figure 8d). The peak R1 corresponds to the insertion of lithium ions into the SWCNT matrix and is more noticeable in the case of P@SWCNTs purified from external phosphorus. The remaining redox peaks are associated with the lithiation/delithiation of phosphorus and the difference in the number of these peaks for P@SWCNTs/P and P@SWCNTs is due to the difference in the structure of phosphorus nanoparticles in these samples [66]. The encapsulated phosphorus has a chain structure and most likely forms certain lithiated alloys. External phosphorus nanoparticles can vary significantly in size and shape and the redox peaks of their reactions with lithium ions overlap, giving broad signals in CV curves (Figure 8a).

The peak current (*i*) is a power-law function of the scan rate (*ν*): *i* = *aν^b^* [67], where *a* and *b* are adjustable parameters. At a value of *b* equal to 0.5, the process has a diffusion character. A *b* value close to 1 indicates that the surface (capacitive) process predominates [8,68,69,70,71,72,73,74]. The value of *b* strongly depends on a number of factors, such as potential, scan rate and charge storage mechanisms [75]. The dependences of log(*i*) on log(*ν*) are straight lines (Figure 8b,e) and the slop of these lines relative to the *x*-axis gives the value of *b* according to the formula log(i) = log(*a*) + *b*log(*ν*). The resulting values for the selected peaks are collected in the inserted tables. All values are greater than 0.5. Therefore, the electrochemical processes occurring in the P@SWCNTs/P and P@SWCNTs electrodes are mainly surface-controlled. The *b* values for the peaks corresponding to the interaction of lithium ions with phosphorus are slightly higher for P@SWCNTs (peaks R2, R3, R4 and O1, O2, O3) as compared to P@SWCNTs/P (peaks R2 and O1, O2). This indicates that the reactions in the P@SWCNTs electrode proceed faster [76].

The contributions of diffusion and capacitive processes to the peak current are separated by the equation: *i* = *k*_1_*ν* + *k*_2_*ν*^1/2^, where the first term corresponds to surface reactions and the second term corresponds to ion intercalation [77,78]. The proportions of these two contributions at different scan rates are displayed in Figure 8c for the P@SWCNTs/P electrode and in Figure 8f for the P@SWCNTs electrode. It can be seen that the capacitive contribution is larger in the case of P@SWCNTs.

## 4. Discussion

To reveal the contribution of SWCNTs to the electrochemical performance of their composites with phosphorus, three samples with different distribution of components were synthesized by means of evaporation and condensation of amorphous red phosphorus.

The SWCNTs/P sample was obtained using closed nanotubes and phosphorus deposited only on the outer surface of the nanotubes. The deposit is significantly oxidized upon contact with laboratory air, as evidenced by the XPS (Figure 4a) and Raman scattering (Figure 5c) data, in which only a small fraction of P-P bonds are detected. These bonds form red phosphorus cores coated with oxidized phosphorus. Interestingly, the SWCNTs/P electrode can operate at a high current density of 5 A g^−1^ for a thousand cycles (Figure 7a). A GDC study of the SWCNTs/P electrode after long-term cycling reveals an insignificant contribution to the capacity from the interaction of lithium ions with SWCNTs (Figure 7b). This is due to the fact that ions cannot penetrate inside capped nanotubes and into the inter-tube space filled with phosphorus. However, SWCNTs ensure the conductivity of the composite electrode and owing to this, the capacity loss is only ~12% at the 1000th cycle.

The P@SWCNTs/P sample was synthesized using pre-opened nanotubes and phosphorus condensed inside the nanotubes and on their surface. The external phosphorus deposit was removed with NaOH solution to obtain P@SWCNTs. The HAADF/STEM analysis showed that the encapsulated phosphorus was retained after the treatment used (Figure 3a–c). According to the HRTEM study, this phosphorus forms long chains inside the nanotubes (Figure 3d).

Surprisingly, the P@SWCNTs/P and P@SWCNTs samples showed very similar specific capacity values (Figure 6f), while the phosphorus content in them differed almost two times according to the XPS and TGA data. We recalculated the obtained values of capacity (C) to the mass of phosphorus determined by the TGA method. The following formula was used for calculation: C(P) = (C(composite) − C(SWCNTs in composite)/(Weight percentage of P), where the C(SWCNTs in composite) was defined as: C(SWCNTs) × Weight percentage of SWCNTs [13,79]. The C(P) values at current densities of 0.1 and 5 A·g^−1^ are 900 and 486 mAh·g^−1^ for P@SWCNTs/P and 1545 and 1006 mAh·g^−1^ for P@SWCNTs. The lower values for P@SWCNTs/P confirm that oxidized external phosphorus, which makes up about 80% of the total phosphorus content, does not contribute to the capacity.

The reported data on the electrochemical performance of P-CNT composites in LIBs are compared with the present results in Table S1. High specific capacities of composite nanomaterials were obtained using multi-walled CNTs [20,29,79,80], which make a significant contribution to the capacity due to the intercalation of lithium ions between nanotube layers. For example, the pristine multi-walled CNTs used to fabricate composite in [44] had a specific capacity of ~500 mAh·g^−1^ at a current density of 0.1 A·g^−1^. This value is double the corresponding value of our o-SWCNTs reference sample. There are only two works on the study of P-SWCNT composites in LIBs [40,81]. Both showed much poorer electrochemical performance as compared to our P@SWCNTs electrode (Table S1). The specific capacity is sometimes given by the mass of the phosphorus component in the composite. The maximum recorded values are 1081 mAh·g^−1^ at 3.9 A·g^−1^ for the ball-milled P-CNT composite [17] and 1264 mAh·g^−1^ at 0.2 A·g^−1^ for the composite obtained from solution [79]. These values do not exceed the corresponding values for our P@SWCNTs sample.

An additional advantage of the P@SWCNTs sample is that it retains a capacity of 93% at 5 A·g^−1^ on the 1000th cycle. The higher rate capability and greater rate retention of P@SWCNTs as compared to P@SWCNTs/P can be explain by two factors. First, the oxidized layer on the surface of external phosphorus particles can slow down the diffusion of lithium ions in the P@SWCNTs/P electrode. Second, lithium ions can reach the encapsulated phosphorus in P@SWCNTs through defects present in the nanotube walls. Indeed, the second and third CV curves of the o-SWCNTs electrode demonstrate a clear redox peak at ~0.8/1.2 V (inset in Figure 6b). Such peaks were previously observed for holey graphene materials with in-plane vacancies 2–5 nm in size [82,83]. These vacancies are likely formed at the stage of SWCNT opening and purification and they are blocked by phosphorus deposits in P@SWCNTs/P. The large accumulation of lithium in P@SWCNTs at high rates (Figure 6f) can only be explained by the presence of channels additional to the open ends of the nanotube. The most probable are vacancy defects of suitable sizes in the sidewalls.

## 5. Conclusions

In summary, we synthesized composite nanomaterials by the vaporization-condensation process using commercial red phosphorus and SWCNTs. Under the synthesis conditions used, phosphorus chains are formed inside open SWCNTs and the SWCNT surface is covered with red phosphorus, which is oxidized upon contact with air. The external phosphorus deposit is effectively removed with an aqueous solution of sodium hydroxide. The content of phosphorus in samples with only external phosphorus SWCNTs/P, only internal phosphorus P@SWCNTs and both types of phosphorus P@SWCNTs/P is 11, 8 and 16 at%, respectively, according to XPS data. To reveal the effect of various combinations of SWCNTs and phosphorus in the composite on the electrochemical interaction with lithium ions, nanomaterials were tested as anodes in coin-cell batteries with lithium sheets as counter electrodes. The SWCNTs/P sample was found to perform better than the reference red phosphorus due to the presence of a conducting network of SWCNTs. Nanotubes practically do not contribute to the SWCNTs/P capacity because their surface is blocked by phosphorus. The creation of composite nanomaterials with internal phosphorus almost doubles the initial Coulombic efficiency as compared to SWCNTs/P and dramatically improves the specific capacity and cycling stability of the electrode. The presence of both external and internal phosphorus leads to a gradual slight degradation of the P@SWCNTs/P electrode over time, associated with slower diffusion processes into the internal volume of the material. The excellent cycling stability of P@SWCNTs over 1000 cycles at a high current density of 5 A·g^−1^ is associated with the synergistic effect of highly capacitive phosphorus and conductive SWCNTs, the absence of inactive oxidized phosphorus deposit and the presence of multiple channels for lithium ion diffusion to encapsulated phosphorus.

## Data Availability

Not applicable.

## Supplementary Materials

The following supporting information can be downloaded at: https://www.mdpi.com/article/10.3390/nano13010153/s1, Figure S1: XRD patterns of P@SWCNTs/P, P@SWCNTs and o-SWCNTs; Figure S2: EDS analysis of P@SWCNTs; Figure S3: XPS survey XPS and P 2p spectra of composite nanomaterials; Figure S4: Ion current curves of evolved gases during decomposition of P@SWCNTs; Figure S5: TG and DTG curves for SWCNTs/P; Figure S6: Experimental and simulated Raman spectra of P@SWCNTs/P; Figure S7: Rate performance of o-SWCNTs and commercial red phosphorus; Table S1: Electrochemical performance of P-CNT composites in lithium-ion batteries, reported in the literature [2,17,20,29,39,40,41,44,79,80,81].
