# Supplementary material for: Single-Walled Carbon Nanotubes with Red Phosphorus in Lithium-Ion Batteries: Effect of Surface and Encapsulated Phosphorus
Source: Nanomaterials (Basel). 2022 Dec 29;13(1):153. doi: 10.3390/nano13010153 (PMC9824809; doi:10.3390/nano13010153)
Supplement: Supplementary file 1 [file nanomaterials-13-00153-s001.zip › nanomaterials-2121483-supplementary.pdf]

Supplementary Materials

# Single-Walled Carbon Nanotubes with Red Phosphorus in Lithium-Ion Batteries: Effect of Surface and Encapsulated Phosphorus

**Anna A. Vorfolomeeva [1], Svetlana G. Stolyarova [1], Igor P. Asanov [1], Elena V. Shlyakhova [1], Pavel E. Plyusnin [1], Evgeny A. Maksimovskiy [1], Evgeny Yu. Gerasimov [2], Andrey L. Chuvilin [3,4], Alexander V. Okotrub [1] and Lyubov G. Bulusheva [1,*]**

[1] Nikolaev Institute of Inorganic Chemistry SB RAS, 3 Acad. Lavrentiev Ave., 630090 Novosibirsk, Russia
[2] Boreskov Institute of Catalysis, SB RAS, 5 Acad. Lavrentiv Ave., 630090 Novosibirsk, Russia
[3] CIC NanoGUNE BRTA, Tolosa Hiribidea 76, E-20018 Donostia-San Sebastián, Spain
[4] IKERBASQUE, Basque Foundation of Science, Maria Diaz de Haro 3, E-48013 Bilbao, Spain
* Correspondence: bul@niic.nsc.ru; Tel.: +73-8333-053-52

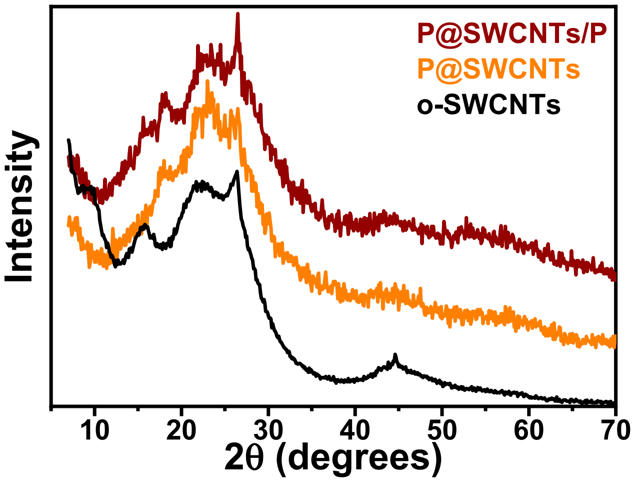


**Figure S1.** XRD patterns measured for initial o-SWCNTs and P@SWCNTs/P, P@SWCNTs samples.

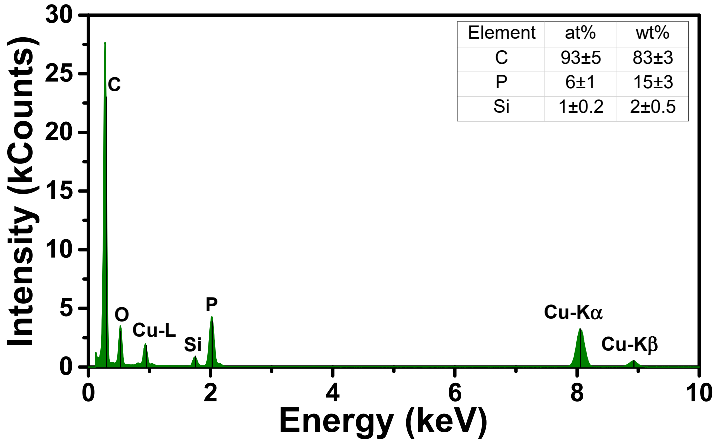


**Figure S2.** EDS analysis of P@SWCNTs sample. The spectrum and content of the main elements (inserted table). Cu and O are associated with the TEM grid, Si is impurity from the quartz ampoule.

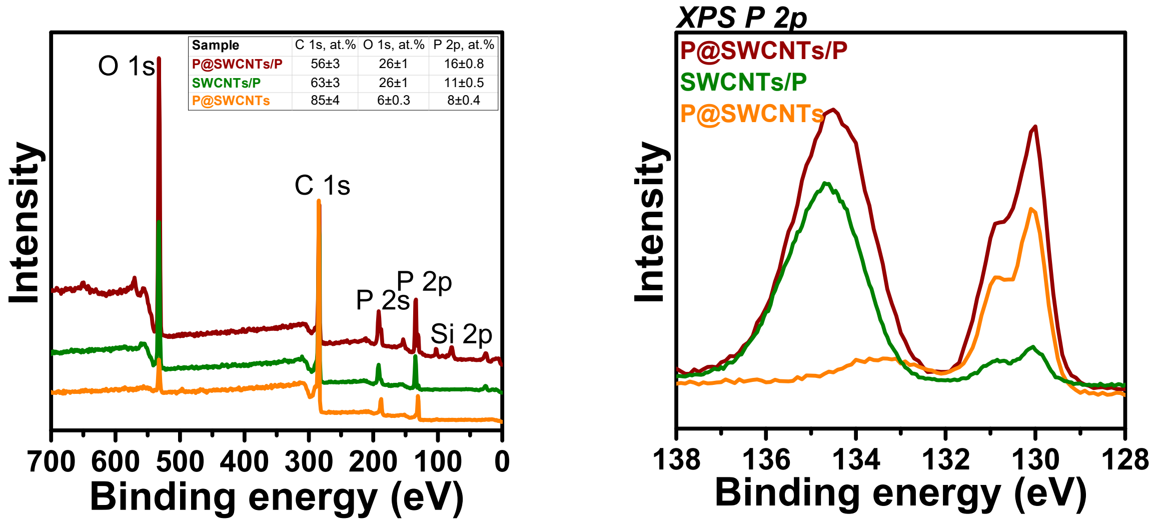


(**a**) (**b**)

**Figure S3.** (**a**) Survey XPS spectra and (**b**) XPS P 2p spectra of P@SWCNTs (encapsulated phosphorus), SWCNTs/P (external phosphorus), and P@SWCNTs/P (encapsulated and external phosphorus). Inset shows the concentrations of carbon, oxygen, and phosphorus in the samples.

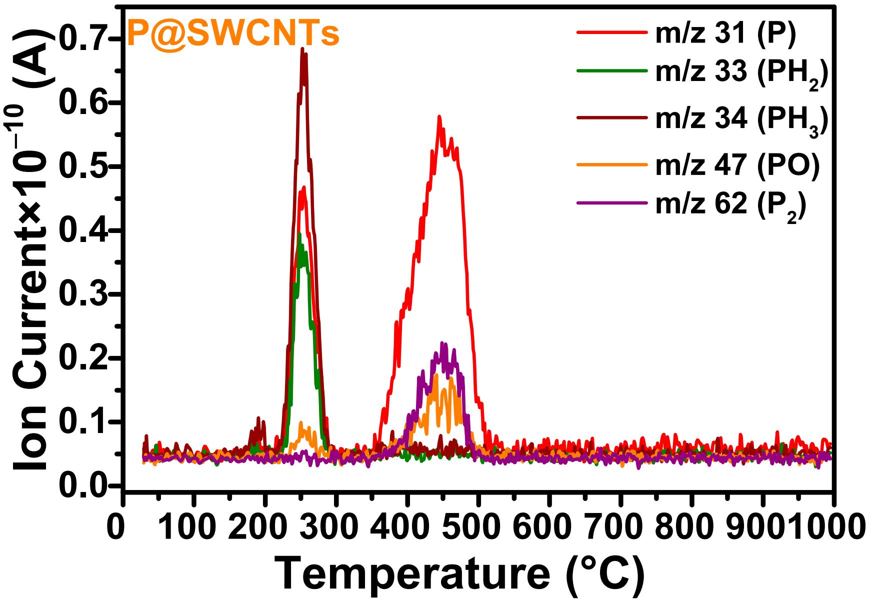


**Figure S4.** Ion current curves of evolved gases during decomposition of P@SWCNTs sample.

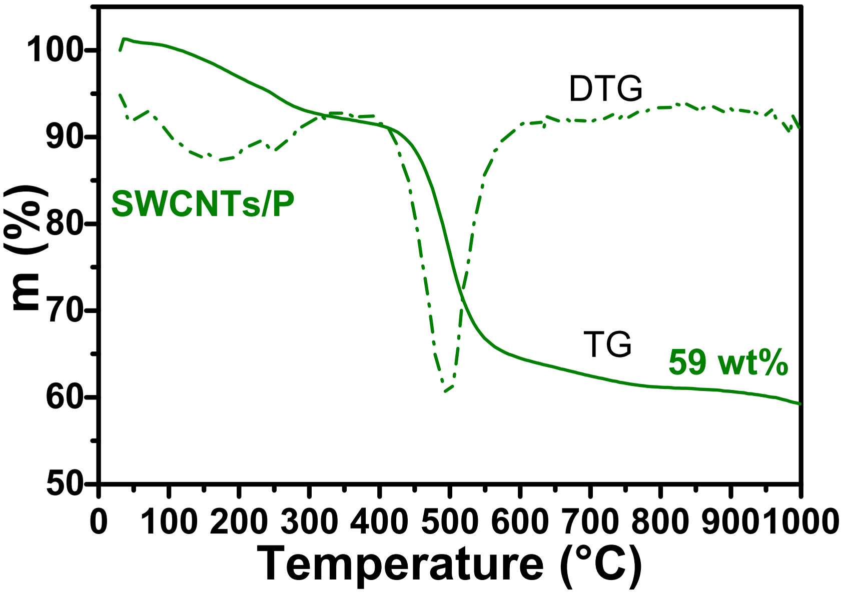


**Figure S5.** TG and DTG curves measured for SWCNTs/P in helium.

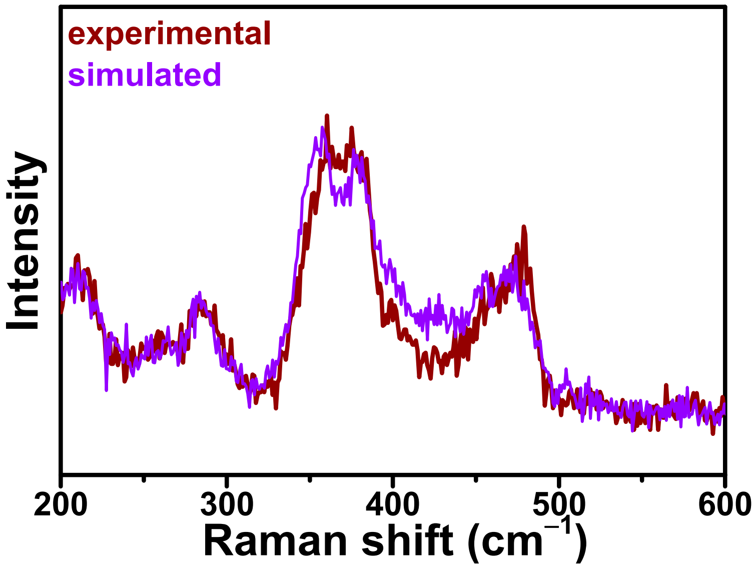


**Figure S6.** Experimental Raman spectrum of P@SWCNTs/P and the spectrum simulated using the spectra of recrystallized phosphorus $P_{re}$ and P@SWCNTs in a ratio of 0.3 to 1.1.

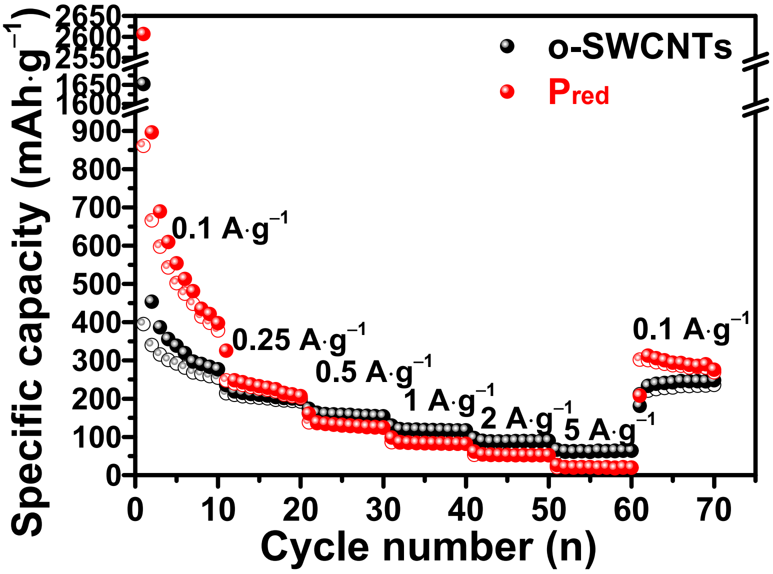


**Figure S7.** Rate performance of o-SWCNTs and commercial red phosphorus ($P_{red}$).

**Table S1.** Electrochemical performance of P–CNT composites in lithium-ion batteries.

| Sample | Synthesis | P content | Current density, A $g^{-1}$ | Specific capacity (mAh $g^{-1}$) of carbon | Specific capacity (mAh $g^{-1}$) based on the composite mass | Specific capacity (mAh $g^{-1}$) based on the phosphorus mass | Cycle performance | Reference |
|---|---|---|---|---|---|---|---|---|
| P-SCNT | vaporization-condensation (vacuum, 700 °C, 4 h) | 36 wt% | 0.1 | ~400 | 1398 | – | 1400 mAh $g^{-1}$ at 100 mA $g^{-1}$ after 200 cycles | 29 |
| | | | 2 | | 782 | – | | |
| P-CNT | ball-milling (Ar, 500 rpm, 40 h) | ? | 0.78 | ~70 | – | 1771 | 1844 mAh $g^{-1}$ at 780 mA $g^{-1}$ after 300 cycles | 17 |
| | | | 3.90 | | – | 1081 | | |
| ARPC-48 | ball-milling (Ar, 450 rpm, 48 h) | ? | 0.016 (0.01 C) | ? | 1994 (не указано) | | 999 mAh $g^{-1}$ at 0.05 C after 50 cycles | 2 |
| | | | 0.8 (0.5 C) | ? | 817 | | | |
| P@CNT | solution-based method, ultrasonicated 5 min at room temperature | 72 wt% | 0.2 | 235–178 | 1082 | 1997–1264 | 736 mAh $g^{-1}$ at 1000 mA $g^{-1}$ after 450 cycles | 79 |
| | | | 10 | – | 784 | – | | |
| P@DMWCNTs | gas-phase synthesis (vacuum, 550 °C, 10 h) | 58 wt% | 0.025 | ~350 | ~500 | 834 | 834 mAh $g^{-1}$ at 25 mA $g^{-1}$ after 50 cycles | 41 |
| PP@MWCNTs | solution-based method (Ar, 189 °C, 8 h) | 32 wt% | 0.1 | ? | – | 886 | 886 mAh $g^{-1}$ at 100 mA $g^{-1}$ after 500 cycles | 39 |
| | | | 3 | ? | – | ~400 | | |
| *r*-P@MWCNTs | vaporization-condensation (vacuum, 500 °C, 48 h, gradient cooling) | 16 wt% | 0.1 | ~500 | 446 | – | 444 mAh $g^{-1}$ at 500 mA $g^{-1}$ after 500 cycles | 44 |
| | | | 3 | ~300 | 246 | – | | |
| RP-on-*r*-P@MWCNTs | vaporization-condensation (vacuum, 500 °C, 48 h, natural cooling) | 72 wt% | 0.1 | ~500 | 780 | – | <444 mAh $g^{-1}$ at 500 mA $g^{-1}$ after 500 cycles | |
| | | | 3 | ~300 | ~700 | – | | |
| P-MWCNTs | wet ball-milling (Ar, 650 rpm, 7 h) | ? | 0.05 | 143 | 1615 | – | 1397 mAh $g^{-1}$ at 50 mA $g^{-1}$ after 50 cycles | 20 |
| | | | 1 | – | 934 | – | | |
| RP NPs/MWNT | solution-based method (100 °C, 2 h) | 56 wt% | 0.2 | ? | 1612 | – | 1577 mAh $g^{-1}$ at 200 mA $g^{-1}$ after 100 cycle | 80 |
| | | | 3 | ? | ~1200 | – | | |
| P–SWCNT | hand-grinding; vaporization-condensation at 600 °C, 2 h | 60 wt% | 0.05 | ~130 at 150 mA/g | 434 | – | 398 mAh $g^{-1}$ at 150 mA $g^{-1}$ after 100 cycles | 40 |
| | | | 1 | – | 160 | – | | |
| SWCNT/P | Hand-grinding, vaporization-condensation at 600 °C, 2h | 70 wt% | 0.05 | – | 800 | – | ~350 mAh $g^{-1}$ at 50 mA $g^{-1}$ on 80th cycle | 81 |
| P@SWCNTs | vaporization-condensation (vacuum, 800 °C, 48 h, natural cooling) | 28 wt% | 0.1 A/g | 245 | 609 | 1545 | 317 mAh $g^{-1}$ at 5000 mA $g^{-1}$ after 1000 cycles | This work |
| | | | 5 A/g | 61 | 328 | 1006 | | |
| P@SWCNTs/P | | 51 wt% | 0.1 A/g | 245 | 578 | 900 | 248 mAh $g^{-1}$ at 5000 mA $g^{-1}$ after 1000 cycles | |
| | | | 5 A/g | 61 | 279 | 486 | | |
